# CSF Concentrations of cAMP and cGMP Are Lower in Patients with Creutzfeldt-Jakob Disease but Not Parkinson's Disease and Amyotrophic Lateral Sclerosis

**DOI:** 10.1371/journal.pone.0032664

**Published:** 2012-03-02

**Authors:** Patrick Oeckl, Petra Steinacker, Stefan Lehnert, Sarah Jesse, Hans A. Kretzschmar, Albert C. Ludolph, Markus Otto, Boris Ferger

**Affiliations:** 1 CNS Diseases Research, Boehringer Ingelheim Pharma GmbH & Co. KG, Biberach an der Riss, Germany; 2 Department of Neurology, University of Ulm, Ulm, Germany; 3 Center for Neuropathology and Prion Research, Ludwig-Maximilians-University Munich, Munich, Germany; Julius-Maximilians-Universität Würzburg, Germany

## Abstract

**Background:**

The cyclic nucleotides cyclic adenosine-3′,5′-monophosphate (cAMP) and cyclic guanosine-3′,5′-monophosphate (cGMP) are important second messengers and are potential biomarkers for Parkinson's disease (PD), amyotrophic lateral sclerosis (ALS) and Creutzfeldt-Jakob disease (CJD).

**Methodology/Principal Findings:**

Here, we investigated by liquid chromatography/tandem mass spectrometry (LC-MS/MS) the cerebrospinal fluid (CSF) concentrations of cAMP and cGMP of 82 patients and evaluated their diagnostic potency as biomarkers. For comparison with a well-accepted biomarker, we measured tau concentrations in CSF of CJD and control patients. CJD patients (n = 15) had lower cAMP (−70%) and cGMP (−55%) concentrations in CSF compared with controls (n = 11). There was no difference in PD, PD dementia (PDD) and ALS cases. Receiver operating characteristic (ROC) curve analyses confirmed cAMP and cGMP as valuable diagnostic markers for CJD indicated by the area under the curve (AUC) of 0.86 (cAMP) and 0.85 (cGMP). We calculated a sensitivity of 100% and specificity of 64% for cAMP and a sensitivity of 67% and specificity of 100% for cGMP. The combination of both nucleotides increased the sensitivity to 80% and specificity to 91% for the term cAMPxcGMP (AUC 0.92) and to 93% and 100% for the ratio tau/cAMP (AUC 0.99).

**Conclusions/Significance:**

We conclude that the CSF determination of cAMP and cGMP may easily be included in the diagnosis of CJD and could be helpful in monitoring disease progression as well as in therapy control.

## Introduction

The cyclic nucleotides cyclic adenosine-3′,5′-monophosphate (cAMP) and cyclic guanosine-3′,5′-monophosphate (cGMP) are important second messengers. Synthesis by adenylate and guanylate cyclases and degradation by phosphodiesterases (PDEs) regulate the concentrations of cAMP and cGMP [1].

In the brain, cAMP and cGMP signaling is involved in a multitude of mechanisms in neurons, astrocytes, oligodendrocytes and microglia. Examples are signal transduction in synapses, communication between neurons and glia cells or inflammatory processes [2], [3]. Alterations of these second messengers affect normal brain function and can be found in several neurological diseases. Parkinson's disease (PD) is characterised by the degeneration of dopaminergic neurons in the substantia nigra. In a rat model of PD, the destruction of nigral dopaminergic neurons with the neurotoxin 6-hydroxydopamine (6-OHDA) resulted in an increase of striatal cAMP and decrease of striatal cGMP [4], [5]. Amyotrophic lateral sclerosis (ALS) is a neurodegenerative disorder characterised by a loss of motor neurons in the spinal cord, brain stem and cerebral cortex. In a genetic mouse model of ALS lower concentrations of cGMP in the cerebral cortex have been reported [6], [7]. In Creutzfeldt-Jakob disease (CJD) a reduced expression of soluble guanylate cyclase β1 in astrocytes in the white matter [8] has been shown but data on cGMP concentrations from human brain samples in CJD are missing.

To date, clinical or even preclinical biomarkers for neurodegenerative diseases such as PD, ALS, CJD are desirable and in the focus of biomedical research [9], [10]. A biomarker as defined by the National Institutes of Health is “a characteristic that is objectively measured and evaluated as an indicator of normal biological processes, pathogenic processes, or pharmacologic responses to a therapeutic intervention” [11]. Cerebrospinal fluid (CSF) is currently the preferred central source for biochemical biomarkers in neurological disorders because CSF is close to the site of the neuropathology and is routinely taken in specialised centers during the clinical work-up of patients under the differential diagnosis of a neurodegenerative disease [12]. However, reliable biomarkers in CSF with the desired degree of specificity and sensitivity for clinical diagnosis of PD, PD dementia (PDD) and ALS are still missing. Usually, post mortem histopathological analysis is necessary to confirm the diagnosis [9], [10], [13]. There are some promising CSF biomarker candidates in PD with α-synuclein as the most important and also in ALS (e.g. neurofilament heavy chain or erythropoietin) but they lack sensitivity and specificity [10], [13]–[17]. In CJD, there are already some CSF markers in use in combination with other diagnostic tools. More precisely, the diagnostic criteria of CJD include the increase in CSF concentrations of the 14-3-3 protein [18]. However, this marker cannot be used in a “screening” situation [19], [20]. Other proteins such as tau, ERK2, ubiquitin or most recently aggregated prion protein (PrP) have been identified as potential biomarkers but their specificity has not yet been confirmed [20]–[23].

The aim of the present study is to investigate cAMP and cGMP in CSF of PD, PDD, ALS, CJD and control patients. The analysis of cAMP and cGMP in CSF is carried out with liquid chromatography coupled to tandem mass spectrometry (LC-MS/MS). To evaluate the suitability of cAMP and cGMP as biomarkers we use receiver operating characteristic (ROC) curves and the Youden index [24] to calculate the cut-off value with the best combination of sensitivity and specificity for diagnosis.

## Materials and Methods

### Patients and CSF collection

All patients included in this study attended to the general outpatient clinic, the outpatient memory clinic, the outpatient clinic for motoneuron diseases or the surveillance unit for transmissible spongiform encephalopathies (University of Göttingen and University of Ulm, Departments of Neurology). The Ethics Committees of the Universities Göttingen and Ulm approved the collection and analysis of CSF samples and specifically approved this study (Ulm). Samples of CJD patients were obtained from the surveillance and therapy trail [20], [25]. All patients or their relatives provided written consent to be included in these studies. CSF was collected by lumbar puncture, centrifuged and stored at −80°C until analysis. The time between lumbar puncture and final storage ranged between 6 and 48 h.

### Patients with PD or PDD

Table 1 shows the characteristics of the PD and PDD patients. The clinical diagnosis including the Mini Mental State Examination (MMSE) and the Hoehn & Yahr score was carried out by neurologists and neuropsychologists according to the DSM-IV criteria and the consensus criteria for PD and PDD [26].

**Table 1:** Characteristics of patients included in the study.

Diagnosis	Gender (m/f)	Age (years)	Disease related characteristics	
**PD study**			**Hoehn & Yahr score**	**MMSE**
CON	4/5	55.4±18.3	-	-
PD	6/5	69.6±9.0	1.9±0.7^a^	27.4±6.4^b^
PDD	2/6	77.9±9.0	3.2±1.4	19.3±7.5^c^
*p*-value		0.005	0.06	0.04
**ALS study**			**First symptoms**	
CON	5/9	49.0±16.8	-	
ALS	9/5	61.4±11.0	S = 8, B = 4, S+B = 2	
*p*-value		0.06		
**CJD study**			***PRNP*** ** Polymorphism** **Codon 129**	**Tau protein (pg/mL)**
CON	6/5	66.4±11.8	not done	300±237
CJD	6/9	60.3±12.9	*MM* = 8, *MV* = 1, *VV* = 2 not done = 4	10188±8477
*p*-value		0.32		0.001

Data are means ± SD, Data are missing for.

a three,

b six and.

c one patients.

CON: control patients, m: male, f: female, S: spinal, B: bulbar, S+B: spinal and bulbar, PRNP: prion protein gene, M: methionine, V: valine.

### Patients with ALS

The group consisted of 14 patients diagnosed with ALS. Table 1 summarises the characteristics of the patients.

### Patients with CJD

Table 1 shows the characteristics of the CJD patients. Nine of the CJD patients were neuropathologically verified as definite CJD cases. Among them, six patients had two methionine alleles (*M/M*) at the *PRNP* codon 129, one was heterozygous (*M/V*) and two patients were homozygous for the valine (*V/V*) allele. Five of the definite cases had a positive 14-3-3 immunoblot and one of them was diagnosed as familial CJD (fCJD). Tau protein exceeded the proposed cut-off of 1300 pg/mL in all but the fCJD case [20]. The genotype at *PRNP* codon 129 of the five neuropathologically-defined probable CJD cases is unknown except for one (*M/M*). All of them had a positive 14-3-3 immunoblot and tau protein in the CSF above the cut-off. A last case underwent no histopathological examination but was diagnosed as fCJD.

### Control patients

We investigated different control groups for the three neurodegenerative diseases to account for age differences. Table 1 shows the characteristics of the groups. The statistical analysis later on showed no difference in the cAMP and cGMP concentrations between the three control groups (*p* = 0.70 for cAMP and *p* = 0.38 for cGMP, Kruskal-Wallis test). The cAMP and cGMP concentrations did not correlate with age as well.

#### Control patients for PD and PDD

The control patients had the following diagnosis: polymyositis (two patients), diabetes mellitus (two patients), amnesia, sinusitis. In three patients the lumbar puncture was carried out to exclude an acute or chronic inflammation.

#### Control patients for ALS

The 14 patients in the control group had the following diagnoses: migraine with aura, episodic headache and symptomatic epilepsy, transient ischemic attack, borreliosis, anterior ischemic optic neuropathy (AION), PD, vestibular neuritis with depression and iron deficiency anemia, pseudotumor cerebri (PTC), migraine and polymyalgia rheumatica. For one of the control patients a lumbar puncture was carried out to exclude an acute or chronic inflammation.

#### Control patients for CJD

The patients in the control group had the following diagnoses: migraine, vestibular neuritis, ALS, vertigo, epilepsy, polyneuropathy, subcortical vascular disease and ischemia, recessive transient attacks, lower body parkinsonism, neuritis retrobulbaris and for one case the lumbar puncture was carried out to exclude an acute or chronic inflammation.

### Determination of tau protein

Total tau protein concentrations in CSF samples of the CJD cohort were measured by a commercially available ELISA (Innogenetics, Gent, Belgium) [20].

### LC-MS/MS analysis of cAMP and cGMP

The CSF samples (35 µL) were mixed with 0.4 M perchloric acid (1∶1) and centrifuged at 20000× g for 10 min at 4°C to precipitate and remove proteins. The supernatant was analysed in duplicate by LC-MS/MS as described previously [27]. The LC-MS/MS system consisted of an HTS PAL autosampler (CTC Analytics AG, Zwingen, Switzerland), maintained at 4°C during all experiments, Agilent 1200 Binary Pump, Agilent 1200 Micro Vacuum Degasser and Agilent 1200 Thermostatted Column Compartment (Agilent Technologies, Morges, Switzerland). Chromatographic separation was carried out at room temperature using a reversed-phase column (Varian MetaSil AQ, 120-5, C18, 100×2.0 mm, Varian, Palo Alto, USA). Mobile phase A consisted of 0.1% formic acid in water and mobile phase B was 100% acetonitrile. The following gradient elution profile was applied at a flow rate of 0.4 mL/min: 0.00 min: 100% A, 0.50 min: 100% A, 1.00 min: 10% A, 2.20 min: 10% A, 2.30 min: 100% A, 3.50 min: 100% A. The column switching valve was set to the waste at 0.00 min, to the mass spectrometer at 1.50 min and to the waste again at 2.50 min.

Eluates were detected with an API 4000 triple quadrupole mass spectrometer (AB Sciex, Ontario, Canada) in the positive electrospray ionisation (ESI) mode by multiple reaction monitoring (MRM). The following transitions were used: 330.08 → 136.10 (cAMP) and 346.15 → 152.10 (cGMP). The injection volume was 25 µL.

Standard solutions were prepared in a mixture (1∶1) of artificial CSF (aCSF, 147 mM NaCl, 2.7 mM KCl, 1.2 mM CaCl_2_, 0.85 mM MgCl_2_, and 1 mM Na_2_HPO_4_, pH 7.0–7.4) and 0.4 M perchloric acid. The stable isotope-labelled internal standards ^13^C_5_-cAMP (transition: 335.15 → 136.20) and ^15^N_5_-cGMP (transition: 351.03 → 157.00) were added to the standard solutions and CSF samples in a concentration of 100 nM to account for matrix effects and variations in ionisation.

### Stability of cAMP and cGMP in CSF

**Figure 1:**
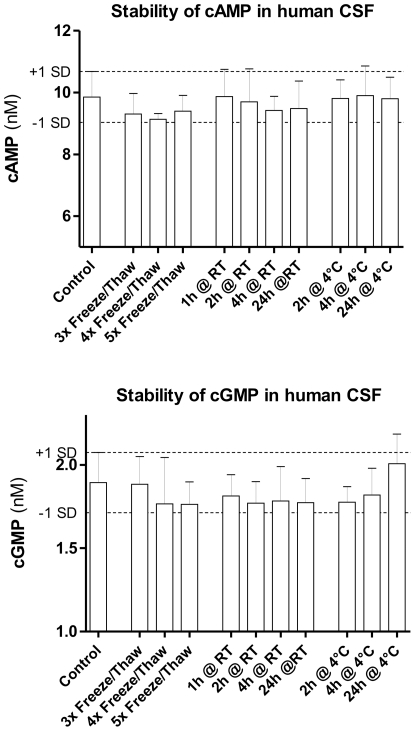
Cyclic AMP and cGMP are stable in human CSF. Stability of cAMP and cGMP in human CSF after different handling and storage conditions. A CSF sample was splitted and subjected to the indicated procedures before being measured by LC-MS/MS in triplicate. Data were analysed by a one-way ANOVA followed by Dunnett's multiple comparison tests to compare the different groups against the control. Data are means ± SD, *p* = 0.58 (cAMP), *p* = 0.70 (cGMP).

A CSF sample was divided into 100 µL aliquots and frozen at −80°C to investigate the stability of cAMP and cGMP. Afterwards the aliquots were either stored at 4°C or at room temperature (RT) for the indicated time (see Fig. 1). Three aliquots underwent a series of freeze/thaw cycles that consisted of thawing at RT, incubating for 5 min at RT and freezing at −80°C.

### Statistics

All statistical analyses were carried out with GraphPad Prism 5.03 (La Jolla, USA). The data of the stability measurement were analysed by a one-way ANOVA and compared with the control sample by Dunnett's multiple comparison test. The cAMP and cGMP concentrations of the different groups of patients were compared by a Mann-Whitney or Kruskal-Wallis test. Spearman's rank correlation coefficient (r) was calculated for cAMP or cGMP with the Hoehn & Yahr score, MMSE, age (PD and PDD), tau protein as well as survival (CJD). A *p*-value<0.05 was regarded as statistically significant. Cut-off levels of cAMP, cGMP, tau protein and tau/cAMP for diagnosis of CJD were selected by calculating the Youden Index of the ROC curves.

## Results

### cAMP & cGMP are stable in human CSF

Different storage and handling conditions during and after CSF collection could affect the results. We checked the stability of cAMP and cGMP in CSF under diverse conditions to reduce the risk of false positive or negative results.

The concentrations measured in the differently treated CSF samples were all within the range of ± 1 SD of the control sample (Fig. 1). This is the intrinsic variation of the LC-MS/MS method. Additionally, we compared CSF samples taken from control subjects and stored for several years or collected recently. There was no significant difference in the mean cAMP and cGMP concentrations (data not shown) indicating the good stability of cAMP and cGMP.

### CSF concentrations of cAMP & cGMP are not different in PD, PDD and controls

**Figure 2:**
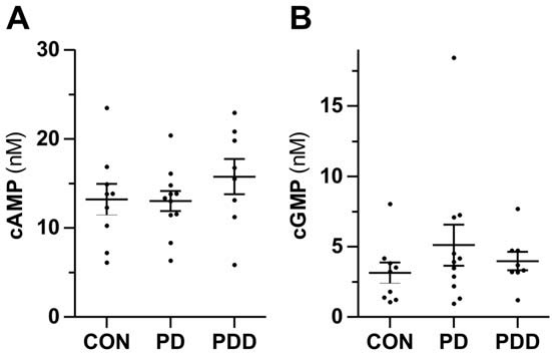
CSF concentrations of cAMP and cGMP are not altered in PD and PDD. CSF concentrations of cAMP (A) and cGMP (B) in cases with Parkinson's disease (PD, n = 11), PD dementia (PDD, n = 8) and control patients (CON, n = 9) measured by LC-MS/MS. Data are means ± SEM, *p* = 0.50 (cAMP), *p* = 0.57 (cGMP), Kruskal-Wallis test.

We measured the concentrations of cAMP and cGMP in CSF samples of patients suffering from PD or PDD and control patients (CON) (Fig. 2). Mean CSF concentrations of cAMP were 13.20 nM (CON), 13.01 nM (PD) and 15.76 nM (PDD) and of cGMP 3.15 nM (CON), 5.11 nM (PD) and 3.97 nM (PDD). There was no statistical significant difference between the groups.

A correlation analysis of the measured CSF concentrations in PD and PDD patients revealed no correlation between cAMP or cGMP and the Hoehn & Yahr score (*p* = 0.80 for cAMP, *p* = 0.70 for cGMP), MMSE (*p* = 0.53 for cAMP, *p* = 0.67 for cGMP) or age (*p* = 0.71 for cAMP, *p* = 0.49 for cGMP).

### CSF concentrations of cAMP & cGMP are not different in ALS and controls

**Figure 3:**
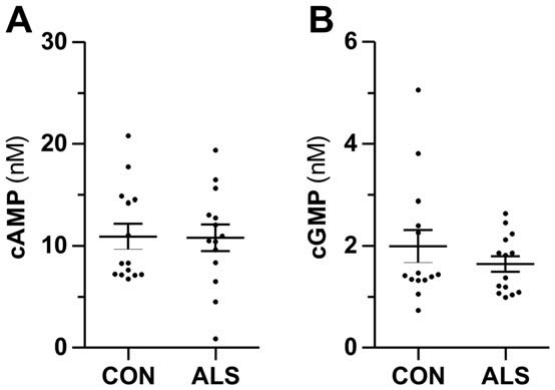
CSF concentrations of cAMP and cGMP are not altered in ALS. CSF concentrations of cAMP (A) and cGMP (B) in cases with amyotrophic lateral sclerosis (ALS, n = 14) and control patients (CON, n = 14) measured by LC-MS/MS. Data are means ± SEM, *p* = 0.80 (cAMP), *p* = 0.48 (cGMP), Mann-Whitney test.

The measurement of the concentrations of cAMP and cGMP in CSF of ALS and control patients yielded mean values of 10.93 nM (CON) and 10.81 nM (ALS) for cAMP and 2.00 nM (CON) and 1.64 nM (ALS) for cGMP (Fig. 3). Neither cAMP nor cGMP concentrations were significantly different between the groups.

### CJD patients have lower CSF concentrations of cAMP & cGMP

**Figure 4:**
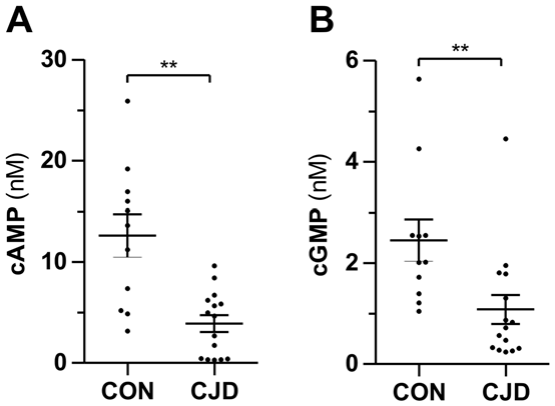
CSF concentrations of cAMP and cGMP are reduced in CJD. CSF concentrations of cAMP (A) and cGMP (B) in cases with Creutzfeldt-Jakob disease (CJD, n = 15) and control patients (CON, n = 11) measured by LC-MS/MS. Data are means ± SEM, **p<0.01, Mann-Whitney test.

The LC-MS/MS analysis of cAMP and cGMP in CSF of patients with CJD showed a marked reduction of cAMP of about 70% compared with controls (Fig. 4). Mean concentrations were 12.60 nM (CON) and 3.90 nM (CJD). We observed a similar decrease for cGMP (−55%) with mean concentrations of 2.45 nM (CON) and 1.08 nM (CJD).

**Figure 5:**
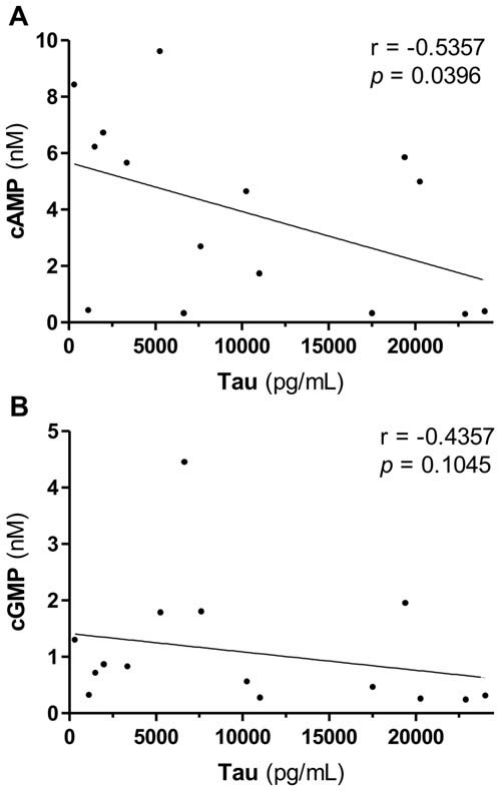
CSF concentrations of cAMP negatively correlate with tau protein in CJD. Correlation of tau protein with cAMP (A) or cGMP (B) concentrations in CSF of Creutzfeldt-Jakob disease (CJD) patients. Spearman's rank correlation coefficient (r) and the respective *p*-values are given.

Since tau protein is suggested for the diagnosis of CJD we looked for a correlation with the cyclic nucleotides [20]. Fig. 5 shows a correlation analysis of cAMP and cGMP with tau protein. There was a weak but significant negative correlation (*p* = 0.04) between cAMP and tau protein concentrations in the CSF of CJD patients (Fig. 5A). Concentrations of cGMP did not correlate with tau protein (Fig. 5B).

We further investigated, whether cAMP and cGMP concentrations correlated with the survival of the CJD patients. The patients had a survival time of 50.9±25.1 d (mean ± SEM) after lumbar puncture. There was no correlation with cAMP (*p* = 0.35) or cGMP (*p* = 0.48).

### Suitability of cAMP & cGMP in CSF as diagnostic biomarkers for CJD

**Figure 6:**
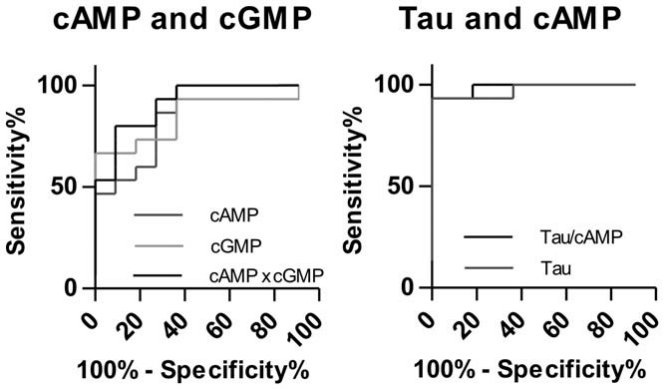
Diagnostic potency of cAMP and cGMP for CJD. Receiver operating characteristic (ROC) curves of cAMP, cGMP and tau protein concentrations in CSF and combinations of them (cAMP×cGMP, tau/cAMP) for the differentiation of Creutzfeldt-Jakob disease (CJD) and control patients. Values for the area under the curve (AUC) and the best combinations of sensitivity and specificity are listed in Table 2.

We calculated ROC curves to evaluate the diagnostic suitability of the cyclic nucleotides in CJD. ROC curves are depicted in Fig. 6 and values for sensitivity, specificity and the area under the ROC curve (AUC) are listed in Table 2. The sensitivity and specificity of cAMP and cGMP increased using the product of the concentrations of cAMP and cGMP.

**Table 2:** Characteristics of potential CSF biomarkers for CJD in ROC analysis.

Parameter	cAMP	cGMP	cAMP×cGMP	Tau protein	Tau/cAMP
AUC	0.8606	0.8545	0.9152	0.9758	0.9879
*p*-value	0.002	0.002	0.001	0.001	0.001
Cut-off^1^	<10.44 nM	<0.96 nM	<5.92	>977 pg/mL	>174.7
Sensitivity (%)	100	66.7	80.0	93.3	93.3
Specificity (%)	63.6	100	90.9	100	100

AUC: area under the curve.

1 The cut-off was calculated using the Youden index [24].

The ratio of tau/cAMP also resulted in an increase of the AUC. Cyclic GMP had the same effect (data not shown).

## Discussion

In this study, we investigated the concentrations of the cyclic nucleotides cAMP and cGMP in CSF of patients suffering from PD, PDD, ALS or CJD in comparison with controls. The measured values for cAMP and cGMP in the control groups agree with previously reported data showing concentrations in the range of 8–14 nM (cAMP) and 2–3 nM (cGMP) [28]–[33]. For the first time we observed significantly lower cAMP and cGMP concentrations in CJD patients. In PD, PDD and ALS patients no effect on cAMP and cGMP CSF concentrations was detected.

Both nucleotides cAMP and cGMP are stable in human CSF during different handling and storage conditions as shown in Fig. 1. Even the storage of CSF over years did not affect cAMP and cGMP concentrations in the present study. The high stability of the cyclic nucleotides in CSF is in agreement with stability measurements in other biological matrices [34].

The impact of the cyclic nucleotides cAMP and cGMP in CSF of PD cases has been questioned for several years [28]–[30], [33], [35]–[37]. Alterations in the central dopaminergic system after pharmacological modulation are reflected in changes of CSF concentrations of cAMP and cGMP [38]. Since dopaminergic neurons in the substantia nigra pars compacta are degenerated in PD, an alteration of CSF cyclic nucleotides seems conceivable. However, in the present study we found no differences in the concentrations of cAMP and cGMP between PD and control patients. This is in line with the findings by Cramer and colleagues who firstly addressed this topic [33] and their observation was confirmed by others [28]–[30], [33], [35]. In contrast, two studies reported reduced cyclic nucleotide concentrations in the CSF of PD patients [36], [37]. A difference in the mean age of the study populations can be ruled-out as a cause for the discrepancies because the concentration of cAMP and cGMP are not age dependent [33]. Drug-induced variations are also unlikely since most of the patients used in the studies were drug-free at the time of CSF collection and L-DOPA, the most widely used anti-parkinsonian drug, does not affect cyclic nucleotides in human CSF [33], [37]. Circadian changes for cAMP do not exist [33]. The discrepancies between some of the studies may be due to the small number of patients and the high inter-individual variations of cAMP and cGMP concentrations.

In contrast to PD, there are no studies of the cyclic nucleotide concentrations in CSF of PDD patients. Similar to the observation in PD patients without dementia we did not find differences in PDD cases. There are reports of alterations in other types of dementia but the findings are not consistent. The discrepancies could be ascribed to distinct primary disorders causing the dementia [39]–[41]. This indicates that the alterations in CSF cAMP and cGMP predominantly originate from other brain regions not responsible for dementia but affected by the primary disease. Our findings support this hypothesis since the cyclic nucleotides were not altered in PD patients without dementia as well.

Two studies investigated CSF cGMP concentrations in ALS patients so far with conflicting results. Ilzecka (2004) showed a 50% decrease of cGMP in CSF of ALS cases but an earlier study by Ikeda et al. (1995) observed no difference [30], [42]. Our results confirm the findings of the study by Ikeda and colleagues that CSF cGMP is not altered in ALS. As discussed by Ilzecka the discrepancy with Ikedas study could be mediated by inclusion of patients with vasomotor headache and increased cGMP concentrations in their control group [42]. In our study no patients with vasomotor headache were included in the control group. Additionally, we examined cAMP concentrations in CSF of ALS patients because it is not known if there are differences in CSF cAMP. Similar to cGMP we observed equal concentrations of cAMP in ALS and control patients. By using a higher number of ALS patients in future studies it would be possible to investigate differences in cAMP and cGMP concentrations between spinal and bulbar onset of ALS as well. Brettschneider and colleagues showed that the type of disease onset may influence CSF concentrations of biomarkers [16]. However, our data indicate that motor neurons which are degenerated in ALS contribute only marginally or not at all to the CSF pool of cAMP and cGMP.

For the first time we investigated concentrations of cAMP and cGMP in CSF of Creutzfeldt-Jakob disease patients. Since there is a substantial degeneration of the whole brain in prion diseases in a relatively short time-span [43], we asked whether this is reflected in CSF cAMP and cGMP. In the present study, CJD patients had about 70% lower cAMP and about 55% lower cGMP concentrations in the CSF than controls. This effect could originate from an increased clearance or decreased synthesis of the nucleotides. Because of the profound degeneration of brain tissue, the decreased synthesis seems to be more likely. There is no concentration gradient between CSF and blood because they contain similar concentrations of cAMP and cGMP [44]. This fact makes an increased diffusion of the cyclic nucleotides into the blood unlikely that could result from an impairment of the blood-CSF-barrier in the choroid plexus. An increased release of PDEs by necrotic cells could also influence the CSF content of cyclic nucleotides.

In our study, the CJD patients distinguish from PD and ALS cases by the reduction of cAMP and cGMP concentrations in CSF. Therefore, we evaluated the suitability of cAMP and cGMP as biomarkers for CJD. The ROC analysis showed that both cyclic nucleotides have the potential for a diagnostic marker. With a sensitivity of 100% and specificity of 63.6% for cAMP and a sensitivity of 66.7% and specificity of 100% for cGMP they do not reach the potency of other CJD biomarkers, e.g. tau protein, 14-3-3 protein or S-100 protein [20], [45], [46]. We obtained a marked increase of the diagnostic potency combining both cyclic nucleotides. With this approach, the sensitivity and specificity (80.0% and 90.9%, respectively) is in the range of other reported CJD biomarkers [46], [47]. Tau protein featured a better diagnostic potential than cAMP and cGMP in our study cohort, but the potency is quite high when being compared with ROC curves of tau protein in other studies [20], [47].

In contrast to cGMP, there is a significant correlation between tau protein and cAMP concentrations. The combination of tau with cAMP in a single ratio tau/cAMP again resulted in an improvement of the diagnostic potency although sensitivity and specificity where similar. We assume that the potential of the combination of tau protein and cAMP is underestimated in our study since the potency of tau protein itself is very high in our cohort. Using cAMP in this ratio seems to be more useful than cGMP because of the more pronounced decrease of cAMP in CJD patients.

Although the diagnostic potency of cAMP and cGMP alone or in combination is lower than for other recently established potential biomarkers of CJD such as ERK2 or ubiquitin, there are still some advantages of the cyclic nucleotides [21], [22]. Both nucleotides are stable in CSF also under different handling and storage conditions. Especially cAMP could be helpful in the differential diagnosis of CJD and Alzheimer's disease (AD) patients because the currently used biomarkers are lacking specificity. Dying neurons release tau protein which indeed is elevated in the CSF of CJD and AD patients. Thus, tau protein is a general marker for neurodegeneration rather than a disease-related biomarker. Similarly, there are AD cases that show a positive result for the 14-3-3 protein test in CSF which is included in the diagnosis of CJD [9]. So far we did not analyse CSF cAMP levels of patients suffering from AD. It has been shown that cAMP increases in the CSF of patients with AD and the cAMP concentrations positively correlate with CSF tau protein [39]. In contrast, our study demonstrates decreased cAMP concentrations in CJD patients and a negative correlation between CSF cAMP and tau protein. These data indicate that cAMP concentrations in the CSF may distinguish between CJD and AD patients. In combination with the very sensitive tau protein determination in CSF, cAMP could improve the specificity of the current diagnostic tests. In the clinic, cAMP and cGMP might be used as markers of disease progression or even as therapy control.

Immunological assays, which are often preferred in the clinic, are commercially available for the determination of cAMP and cGMP if LC-MS/MS analysis is not possible. They enable a simple and fast analysis without involving expensive equipment. However, Zhang and colleagues (2009) showed the advantage of LC-MS/MS over enzyme based immuno assays by comparing a competitive enzyme immunoassay (EIA) for the analysis of cGMP in plasma samples and a LC-MS/MS assay for the same analytes. The LC-MS/MS method showed a better precision and accuracy as well as reduced matrix effects [34]. In our study the LC-MS/MS method used for the analysis of the cyclic nucleotides features the convenience of measureing cAMP and cGMP simultaneously combined with the provided high selectivity of LC-MS/MS.

The combination of cAMP and cGMP or tau protein and cAMP is a good example for the combination of different markers to obtain a higher diagnostic potency. This is important since biomarkers related to the pathological process are not always available. Hence, “secondary” markers that indicate downstream effects of the pathological events have to be used. However, frequently they lack specificity for the disease as described above for tau protein. Such weaknesses could be solved or reduced by combinations of biomarkers. In this context, LC-MS/MS analysis is a beneficial tool as shown in the present study. It allows the simultaneous measurement of several analytes in a single run.

### Conclusion

In conclusion, we showed reduced concentrations of cAMP and cGMP in CSF of CJD patients but not in PD, PDD or ALS. This may be owing to the widespread brain atrophy in CJD compared with the localised neurodegeneration in the other diseases. Especially in combination, cAMP and cGMP have a diagnostic potential for CJD. In combination with tau protein, cAMP led to a further improvement of this marker. Since there are already fast and reliable immunological assays for cAMP and cGMP available the cyclic nucleotides can easily be included into routine analysis and may improve the diagnosis of CJD if LC-MS/MS is not applicable. The measurement of cAMP and cGMP CSF concentrations may be useful to monitor disease progression and therapy control. Our study also highlighted the advantages of LC-MS/MS for the combination of different biomarkers.
